# Selective IgA Deficiency and COVID-19 Outcomes: A Nationwide Retrospective Cohort Study

**DOI:** 10.3390/jcm15072487

**Published:** 2026-03-24

**Authors:** Rawi Hazzan, Nur Abu Ahmad, Mifleh Tatour, Naiel Bisharat, Ziv Neeman

**Affiliations:** 1 Clalit Health Services, Afula 1834111, Israel; ravih@clalit.org.il (R.H.); nur.abu-ahmad@biu.ac.il (N.A.A.); 2 Emek Medical Center, Afula 1834111, Israel; bisharat_na@clalit.org.il (N.B.); ziv_ne@clalit.org.il (Z.N.)

**Keywords:** selective IgA deficiency, SARS-CoV-2 infection, mucosal immunity, COVID-19 outcomes, vaccination response

## Abstract

**Background:** Selective immunoglobulin A deficiency (sIgAD), the most common primary immunodeficiency, is associated with recurrent respiratory infections. Despite the established role of IgA in mucosal immunity, population-based data evaluating COVID-19 susceptibility and severity among individuals with sIgAD are scarce. **Objectives:** This study aimed to evaluate the association between selective IgA deficiency and the risk of SARS-CoV-2 infection, recurrent infection, COVID-19-related hospitalization, and vaccination uptake. Design and Setting: We conducted a retrospective population-based cohort study using the Clalit Health Services electronic health record database in Israel. **Methods:** Adults aged ≥18 years with documented serum IgA measurements between 2020 and 2022 were included. Selective IgA deficiency was defined as serum IgA < 7 mg/dL with normal IgG and IgM levels. Individuals with sIgAD were matched 1:4 with controls with normal IgA levels by age and sex. Outcomes included documented SARS-CoV-2 infection, recurrent infection (>2 episodes), COVID-19-related hospitalization, and vaccination status. Multivariable logistic regression models were adjusted for demographic characteristics, comorbidities, and vaccination status. **Results:** The matched cohort included 61,150 individuals (12,230 with sIgAD and 48,920 controls). The risk of primary SARS-CoV-2 infection did not differ significantly between groups (13.0% vs. 14.0%; adjusted OR 1.03, 95% CI 0.95–1.12). However, individuals with sIgAD had increased odds of recurrent infection (adjusted OR 1.15, 95% CI 1.09–1.22) and COVID-19-related hospitalization (adjusted OR 1.40, 95% CI 1.22–1.60). Booster vaccination uptake was slightly higher among individuals with sIgAD. **Conclusions:** Selective IgA deficiency was not associated with increased susceptibility to primary SARS-CoV-2 infection but was independently associated with recurrent infection and increased risk of hospitalization. These findings underscore the importance of mucosal immunity in post-infection viral control and suggest that individuals with sIgAD may benefit from closer monitoring after COVID-19 infection.

## 1. Introduction

The coronavirus disease 2019 (COVID-19) pandemic, caused by severe acute respiratory syndrome coronavirus 2 (SARS-CoV-2), emerged in December 2019 and rapidly evolved into a global health crisis [1]. Previous zoonotic coronavirus outbreaks, including SARS-CoV in 2002 and MERS-CoV in 2012, were characterized by high case-fatality rates of approximately 10% and 34%, respectively [2]. In contrast, the COVID-19 outbreak had a lower case-fatality rate, but its unprecedented transmissibility resulted in millions of infections worldwide and substantial morbidity and mortality [3]. Viral entry is mediated by binding of the SARS-CoV-2 spike protein to the angiotensin-converting enzyme 2 (ACE2) receptor, which is abundantly expressed in the respiratory epithelium, thereby facilitating infection and replication [1].

Humoral immune responses following SARS-CoV-2 infection typically begin with the appearance of IgM antibodies directed against viral antigens, followed by class-switch recombination to IgG and, importantly, to IgA in mucosal tissues [4,5]. Immunoglobulin A (IgA) represents the dominant antibody isotype at mucosal surfaces and accounts for the majority of total immunoglobulin production in humans [6,7]. IgA exists in monomeric form in serum and dimeric secretory form at mucosal interfaces, where it plays a critical role in immune exclusion, viral neutralization, and prevention of pathogen adherence to epithelial cells [7,8]. In respiratory viral infections, secretory IgA constitutes a first line of defense by limiting viral replication in the upper airways and shaping local immune memory.

Selective IgA deficiency (sIgAD), defined as serum IgA levels < 7 mg/dL in individuals older than four years with normal IgG and IgM levels, is the most common primary immunodeficiency [9]. Although many individuals with sIgAD remain asymptomatic, approximately 20–30% experience recurrent sinopulmonary and gastrointestinal infections, as well as increased prevalence of autoimmune and allergic disorders [9,10]. The absence of mucosal IgA raises the possibility of impaired protection against respiratory pathogens, including SARS-CoV-2.

Accumulating evidence has highlighted the central role of IgA responses in COVID-19 pathogenesis and recovery; several clinical and immunologic studies have demonstrated that mucosal and systemic IgA responses correlate with disease severity and viral clearance [4,5,11,12,13,14,15]. Altered IgA response patterns have been observed in both severe and mild COVID-19, and persistent abnormalities have been described in long COVID syndromes [11,12,13]. Moreover, elevated IgA and antiphospholipid IgA titers have been associated with severe disease and thrombotic complications [16,17]. At the same time, ecological observations suggest lower infection and mortality rates in regions with very low IgA deficiency prevalence [18]. Furthermore, vaccine-induced mucosal IgA responses appear to decline over time and may not mirror the robust mucosal responses observed after natural infection [18,19,20].

Beyond antibody responses, host-related factors such as smoking, chronic lung disease, sex, and age influence an individual’s susceptibility to COVID-19 and disease severity [16,21,22,23,24]. These factors may interact with mucosal immune competence and further modulate clinical outcomes.

Despite increasing recognition of the importance of mucosal immunity in SARS-CoV-2 infection, population-level data directly comparing COVID-19 susceptibility and clinical outcomes between individuals with and without selective IgA deficiency remain scarce. No large retrospective cohort study has comprehensively evaluated infection rates, reinfection patterns, hospitalization risk, and vaccination uptake in patients with laboratory-confirmed sIgAD within a real-world healthcare system.

Therefore, this study aimed to investigate the association between selective IgA deficiency and the risk of SARS-CoV-2 infection, recurrent infection, COVID-19-related hospitalization, and vaccination uptake in a large nationwide population-based cohort.

## 2. Methods

This retrospective, population-based cohort study was conducted using the centralized electronic health record (EHR) database of Clalit Health Services (CHS), the largest integrated healthcare organization in Israel, serving approximately 4.8 million members, representing about 52% of the national population. The CHS EHR includes comprehensive longitudinal data on demographics, outpatient and inpatient diagnoses, procedures, laboratory tests, medications, and vaccinations, with near-complete capture of healthcare encounters across primary, specialty, and hospital settings. The study period extended from 1 January 2020, to 31 December 2022, encompassing multiple waves of the COVID-19 pandemic and the national vaccination campaign. All data were retrieved using structured queries and de-identified before analysis in accordance with local data protection regulations.

### 2.1. Study Population and Exposure Definition

We identified all adult CHS members aged 18 years or older who had at least one documented serum IgA measurement obtained during routine clinical care or diagnostic evaluation between 1 January 2020, and 31 December 2022.

Serum IgA testing in routine clinical practice is typically performed during evaluation of suspected immunodeficiency, recurrent respiratory or gastrointestinal infections, autoimmune disorders (e.g., celiac disease), or broader immunologic workups. For each individual, the index IgA value was defined as the earliest available measurement during this period. Selective IgA deficiency (sIgAD) was defined according to international criteria as a serum IgA level < 7 mg/dL in individuals older than four years, in the presence of normal serum IgG and IgM levels measured within six months of the index IgA measurement.

Individuals with documented diagnoses of common variable immunodeficiency, severe combined immunodeficiency, or other major primary immunodeficiency syndromes, identified using ICD-10 codes and immunology clinic records, were excluded. Patients with missing key demographic variables (age or sex) or incomplete follow-up were also excluded. The comparison group consisted of adults with serum IgA levels within the laboratory reference range, normal IgG and IgM levels, and no documented diagnosis of primary immunodeficiency. Exposure status was treated as a fixed baseline characteristic.

### 2.2. Outcomes

Outcomes were ascertained using CHS electronic health records and integrated national registries. Primary SARS-CoV-2 infection was defined as at least one documented positive SARS-CoV-2 RT-PCR or antigen test during the study period. Recurrent infection was defined as more than 2 COVID-19 infections, with at least 60 days between positive test episodes to reduce misclassification due to prolonged viral shedding.

COVID-19-related hospitalization was defined as an acute hospital admission with a principal or secondary diagnosis of COVID-19 (ICD-10 codes U07.1 or U07.2), or hospitalization occurring within 14 days of a positive SARS-CoV-2 test. Additional hospitalization outcomes included the number of COVID-19-related admissions and the mean length of hospital stay per admission.

COVID-19 vaccination status was obtained from the national immunization registry and CHS records and categorized by the number of doses received (0, 1, 2, or more than 2). All vaccine types included in the national vaccination program were considered.

### 2.3. Covariates

Baseline covariates were extracted at the index date. They included age, sex, and body mass index (BMI), calculated using the most recent measurement within one year before the index date. Comorbidities, including diabetes mellitus, hypertension, ischemic heart disease, chronic kidney disease, chronic obstructive pulmonary disease, and malignancy, were identified using validated ICD-10 code algorithms and medication proxies. Vaccination status was also included as a covariate in multivariable models.

### 2.4. Matching Procedure

To minimize confounding by age and sex, individuals with sIgAD were matched with up to four individuals with normal IgA levels using propensity score matching in a 4:1 ratio. Propensity scores were estimated using logistic regression, including age and sex as predictors. Age and sex were selected because they are among the strongest demographic determinants of COVID-19 outcomes. Matching was performed using nearest-neighbor matching with a caliper width of 0.2 standard deviations of the logit of the propensity score, consistent with the recommended. Baseline balance between groups was assessed after matching, and only matched individuals were included in the final analytic cohort.

### 2.5. Statistical Analysis

Categorical variables were summarized as counts and percentages, and continuous variables as mean ± standard deviation or median with interquartile range, as appropriate. Between-group comparisons were conducted using the χ^2^ test for categorical variables and the independent-samples *t* test or Mann–Whitney U test for continuous variables.

Multivariable logistic regression models were used to estimate odds ratios (ORs) and 95% confidence intervals (CIs) for the association between selective IgA deficiency and study outcomes. Models were adjusted for age, sex, BMI, major comorbidities, and COVID-19 vaccination status. A two-sided *p*-value < 0.05 was considered statistically significant. All analyses were performed using SPSS version 28.0 (IBM Corp., Armonk, NY, USA) and R version 4.1.1 (R Foundation for Statistical Computing, Vienna, Austria). 

While preparing this work, the authors used Grammarly Pro software to improve readability and language. After using this tool/service, the authors reviewed and edited the content as needed and took full responsibility for the publication’s content.

### 2.6. Follow-Up and Sensitivity Analyses

The index date was defined as the date of the first recorded IgA measurement. Participants were followed from the index date until the occurrence of the outcome of interest, death, disenrollment from CHS, or 31 December 2022, whichever occurred first.

Sensitivity analyses were conducted to evaluate the robustness of the findings, including restriction to matched cohorts and subgroup analyses. Laboratory measurements were performed in CHS central laboratories using standardized nephelometric or turbidimetric methods, and for individuals with multiple measurements, the earliest available value was used.

## 3. Results

### 3.1. Study Population

After applying inclusion and exclusion criteria and performing 4:1 matching by age and sex, the final cohort comprised 61,150 adults: 12,230 with selective IgA deficiency and 48,920 with normal IgA levels. The mean age of the study population was 60.0 ± 20.9 years, and 56.5% were male.

Baseline demographic and clinical characteristics were generally well balanced between the groups after matching, as summarized in Table 1. There were no clinically meaningful differences in major comorbidities or BMI distributions between individuals with sIgAD and those with IgA levels within the normal range.

### 3.2. COVID-19 Infection and Reinfection

The overall incidence of documented SARS-CoV-2 infection during the study period was similar in the two groups: 13.0% (1590/12,230) among individuals with sIgAD and 14.0% (6859/48,920) among those with normal IgA levels. In multivariable logistic regression, sIgAD was not associated with a higher risk of primary infection compared with normal IgA levels (adjusted OR 1.03, 95% CI 0.95–1.12; *p* = 0.44), indicating no significant difference in susceptibility to initial SARS-CoV-2 infection.

**Figure 1:**
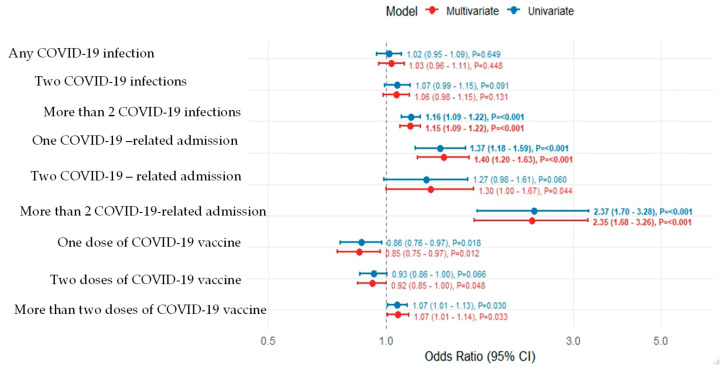
Odds ratios for COVID-19 outcomes comparing individuals with normal IgA levels and those with selective IgA deficiency. Unadjusted and adjusted logistic regression models are shown. Error bars represent 95% confidence intervals. The vertical dashed line at an odds ratio of 1 indicates no difference between groups.

Among participants with at least one infection, the median interval between recurrent infections was comparable between groups: 88.4 days (interquartile range 4.7–339.2) in the sIgAD group and 82.9 days (5.0–338.9) in the normal IgA group (*p* = 0.49). However, recurrent infections (more than two documented episodes) were more frequent among individuals with sIgAD, occurring in 7109 of 12,230 participants (58.1%) compared with 26,976 of 48,920 (55.2%) in the normal IgA group. This difference remained statistically significant in the adjusted analysis (adjusted OR 1.15, 95% CI 1.09–1.22; *p* < 0.001), suggesting an increased propensity for reinfection among IgA-deficient individuals (Table 2, Figure 1).

### 3.3. Hospitalization Outcomes

COVID-19-related hospitalization was relatively uncommon overall but occurred more often in the sIgAD group. During follow-up, 3.1% of individuals with sIgAD were hospitalized at least once for COVID-19, compared with 2.2% of those with normal IgA (*p* < 0.001). In multivariable models, sIgAD was associated with a significantly increased odds of COVID–19-related hospitalization (adjusted OR 1.40, 95% CI 1.22–1.60; *p* < 0.001). There were no COVID-19-related hospitalizations for 96.9% and 97.8% of the sIgAD and normal IgA groups, respectively (*p* < 0.001). Single admissions occurred in 2.0% of the sIgAD group versus 1.5% of the normal IgA group; two admissions in 0.7% versus 0.5%; and more than two admissions in 0.4% versus 0.2%, respectively (all *p* < 0.001). These findings indicate a higher burden of repeated hospitalizations among patients with IgA deficiency (Table 3). The mean length of stay per COVID-19-related hospitalization was 4.39 ± 4.23 days in participants with sIgAD and 6.2 ± 6.56 days in those with normal IgA (*p* = 0.03). Although hospital stays were slightly shorter among IgA-deficient patients, this was offset by their higher likelihood of initial and repeated admissions.

### 3.4. Vaccination Status

COVID-19 vaccination coverage was high in both groups. In the sIgAD group, 14.0% had received no vaccine doses, 3.1% had received 1 dose, 10.5% had received 2 doses, and 72.4% had received more than 2 doses. In the normal IgA group, the corresponding proportions were 14.5%, 3.8%, 11.7%, and 70.1%, respectively.

Individuals with sIgAD were slightly more likely to have received more than two vaccine doses than controls (72.4% vs. 70.1%; adjusted OR 1.07, 95% CI 1.01–1.13; *p* = 0.03), consistent with slightly higher booster vaccination uptake in this population (Table 4). Despite this, the increased risks of recurrent infection and hospitalization in the sIgAD group persisted, suggesting that standard systemic vaccination alone may not fully compensate for the absence of mucosal IgA.

## 4. Discussion

### 4.1. Summary

In this large population-based retrospective cohort study, selective IgA deficiency was not associated with an increased risk of documented primary SARS-CoV-2 infection. However, individuals with sIgAD demonstrated a significantly higher likelihood of recurrent infections and COVID-19-related hospitalization. These findings suggest that while systemic immune mechanisms and vaccination may provide sufficient protection against initial infection, the absence of mucosal IgA may impair effective viral control and resolution, thereby predisposing patients to recurrent disease and more complicated clinical courses.

### 4.2. Comparison with the Existing Literature

IgA plays a pivotal role in mucosal immune defense by neutralizing respiratory viruses at the epithelial surface and preventing viral attachment and replication [6,7,8,25]. Clinical studies have consistently demonstrated that robust mucosal IgA responses are associated with lower viral loads and improved outcomes in COVID-19 [4,5,11,14,15]. Conversely, dysfunctional or dysregulated IgA responses have been linked to severe disease and fatal outcomes [16,17].

Our findings extend these observations by providing epidemiologic evidence that the absence of IgA does not substantially increase susceptibility to primary infection but is associated with impaired control following exposure. The increased rate of recurrent infections observed in individuals with sIgAD is biologically plausible, as secretory IgA contributes to immune exclusion and local immune memory in the respiratory tract. Without effective mucosal neutralization, repeated viral replication at the epithelial interface may occur despite intact systemic IgG responses.

Ecological data suggesting a lower COVID-19 burden in countries with very low IgA deficiency prevalence [18,25] should be interpreted cautiously, given potential confounding. However, our individual-level matched analysis supports the concept that IgA contributes more strongly to post-exposure control and disease modulation than to absolute prevention of viral acquisition.

### 4.3. Disease Severity and Healthcare Utilization

Although absolute hospitalization rates were low in both groups, individuals with sIgAD were significantly more likely to require hospitalization and multiple admissions. This pattern indicates increased disease complexity rather than merely increased infection frequency. Possible mechanisms include impaired early viral containment, prolonged viral shedding, altered inflammatory regulation at mucosal surfaces, and increased vulnerability to secondary bacterial infections.

Interestingly, the mean length of hospital stay was slightly shorter among IgA-deficient patients, suggesting that, once hospitalized, the disease trajectory may be influenced by systemic immune factors beyond IgA-mediated mechanisms. Nevertheless, the overall increase in admissions underscores the clinical significance of morbidity.

### 4.4. Vaccination and Reinfection

Vaccination uptake was high in both groups, with slightly higher rates among individuals with sIgAD. However, enhanced vaccination coverage did not eliminate the increased risk of recurrent infection and hospitalization. This observation aligns with studies demonstrating that intramuscular vaccination induces strong systemic IgG responses but relatively limited and transient mucosal IgA responses [17,19]. These findings underscore the potential importance of mucosal-targeted vaccine strategies or adjunctive preventive approaches in populations with impaired IgA-mediated immunity.

### 4.5. Strengths and Limitations

The strengths of this study include its large sample size, nationwide real-world data, laboratory-defined selective IgA deficiency, and comprehensive adjustment for demographic variables, comorbidities, and vaccination status. Including recurrent infection and hospitalization as outcomes provide clinically relevant insight beyond simple infection incidence.

Several limitations merit consideration. The retrospective observational design precludes causal inference; asymptomatic infections may have been underdiagnosed, and behavioral factors, occupational exposure, and adherence to mitigation measures were not available for analysis. Requiring a documented serum IgA measurement may have introduced selection bias by preferentially including individuals with higher healthcare utilization. Because individual-level data on testing frequency were not available, we cannot fully exclude the possibility of ascertainment bias. Additionally, COVID-19-related hospitalization was defined as admission within 14 days of a positive SARS-CoV-2 PCR test or with a principal or secondary COVID-19 diagnosis, which may include incidental infections and lead to possible outcome misclassification. Variant-specific effects and direct immunologic measurements were not assessed, limiting mechanistic interpretation.

### 4.6. Clinical Implications and Future Directions

These findings have practical implications for clinicians caring for patients with selective IgA deficiency. While intensified preventive measures may not be necessary to reduce the risk of primary infection, heightened vigilance following SARS-CoV-2 infection is warranted. Early reassessment, close follow-up, and timely antiviral therapy may be particularly important in this population to mitigate recurrent disease and the risk of hospitalization.

Future research should focus on the detailed characterization of mucosal immune responses in IgA-deficient individuals, the evaluation of mucosal vaccination strategies, and the investigation of long-term outcomes following repeated SARS-CoV-2 exposure. Understanding the interaction between systemic and mucosal immunity may inform the development of optimized prevention strategies for individuals with primary immunodeficiencies.

## Figures and Tables

**Table 1:** Baseline demographic and clinical characteristics of participants with selective IgA deficiency and matched controls with normal IgA levels. Values are presented as mean ± standard deviation or number (percentage).

	IgA Deficiency	Normal IgA	*p* Value
n	12,230	48,920	
Age (Mean SD)	60.0 (20.9)	60.0 (20.9)	1
Gender n (%)			
Female	5321 (43.5)	21,284 (43.5)	1
Male	6909 (56.5)	27,636 (56.5)	1
**Chronic diseases n (%)**			
Malignancy	2162 (17.7)	8628 (17.6)	0.926
s/p CVA	960 (7.8)	3718 (7.6)	0.363
IHD	1805 (14.8)	7173 (14.7)	0.799
CHF	856 (7.0)	3457 (7.1)	0.81
Hypertension	4386 (35.9)	17,570 (35.9)	0.921
Intellectual disability	81 (0.7)	343 (0.7)	1
CRF	1168 (9.6)	4572 (9.3)	0.499
Cirrhosis	145 (1.2)	634 (1.3)	0.438
Chronic Hepatitis	152 (1.2)	595 (1.2)	0.847
Other Liver Disease	323 (3.0)	1389 (2.8)	0.463
AIDS/HIV	21 (0.2)	68 (0.1)	0.474

Abbreviations: CVA, cerebrovascular accident; IHD, ischemic heart disease; CHF, congestive heart failure; CRF, chronic renal failure; AIDS, acquired immunodeficiency syndrome; HIV, human immunodeficiency virus; s/p, status post. Intellectual disability was defined according to ICD-10 codes F70–F79.

**Table 2:** SARS-CoV-2 infection outcomes among individuals with selective IgA deficiency and those with normal IgA levels. Recurrent infection was defined as more than 2 COVID-19 infections. The interval between infections is presented as the median (interquartile range).

Outcome	IgAD n = 12,230	Normal IgA n = 48,920	*p*-Value
Any COVID-19 infection, n (%)	1590 (13.0)	6859 (14.0)	<0.001
Interval between recurrent infections, days	88.4 (4.7–339.2) #	82.9 (5.0–338.9) #	0.49
Recurrent infections (>2 episodes), n (%)	7109 (58.1)	26,976 (55.2)	<0.001

# Median (IQR).

**Table 3:** COVID-19-related hospitalization frequency and length of hospital stay among individuals with selective IgA deficiency and those with normal IgA levels.

Number of COVID Admissions	IgA-Deficient (N = 12,230)	Normal IgA (N = 48,920)	*p*-Value
0	11,847 (96.9%)	47,843 (97.8%)	<0.001
1	242 (2.0%)	712 (1.5%)	<0.001
2	84 (0.7%)	268 (0.5%)	<0.001
>2	57 (0.4%)	97 (0.2%)	<0.001
Mean length of hospitalization, days (SD)	4.39 (4.23)	6.2 (6.56)	0.03

**Table 4:** COVID-19 vaccination status among individuals with selective IgA deficiency and those with normal IgA levels. Values are presented as numbers (percentages).

Number of Vaccinations	IgAD (N = 12,230)	Normal IgA (N = 48,920)	*p*-Value
0	1714 (14.0%)	7080 (14.5%)	<0.001
1	384 (3.1%)	1838 (3.8%)	<0.001
2	1283 (10.5%)	5715 (11.7%)	<0.001
>2	8849 (72.4%)	34,287 (70.1%)	<0.001

Values are n (%).

## Data Availability

The data supporting the findings of this study are not publicly available due to privacy and ethical restrictions. However, they are available from Clalit Health Services upon reasonable request and subject to institutional approval.
